# Regulatory Aspects of the Vacuolar CAT2 Arginine Transporter of *S. lycopersicum*: Role of Osmotic Pressure and Cations

**DOI:** 10.3390/ijms20040906

**Published:** 2019-02-19

**Authors:** Jessica Cosco, Teresa M. R. Regina, Mariafrancesca Scalise, Michele Galluccio, Cesare Indiveri

**Affiliations:** 1 Department DiBEST (Biologia, Ecologia, Scienze della Terra), Unit of Biochemistry and Molecular Biotechnology, University of Calabria, Via P. Bucci 4C, 87036 Arcavacata di Rende, Italy; jessica.cosco@unical.it (J.C.); teresa.regina@unical.it (T.M.R.R.); mariafrancesca.scalise@unical.it (M.S.); michele.galluccio@unical.it (M.G.); 2 CNR Institute of Biomembranes, Bioenergetics and Molecular Biotechnology, via Amendola 165/A, 70126 Bari, Italy

**Keywords:** cholesterol, arginine, osmolyte, vacuole, transport, protein expression, CRAC

## Abstract

Many proteins are localized at the vacuolar membrane, but most of them are still poorly described, due to the inaccessibility of this membrane from the extracellular environment. This work focused on the characterization of the CAT2 transporter from *S. lycopersicum* (*Sl*CAT2) that was previously overexpressed in *E. coli* and reconstituted in proteoliposomes for transport assay as [^3^H]Arg uptake. The orientation of the reconstituted transporter has been attempted and current data support the hypothesis that the protein is inserted in the liposome in the same orientation as in the vacuole. *Sl*CAT2 activity was dependent on the pH, with an optimum at pH 7.5. *Sl*CAT2 transport activity was stimulated by the increase of internal osmolality from 0 to 175 mOsmol while the activity was inhibited by the increase of external osmolality. K^+^, Na^+^, and Mg^2+^ present on the external side of proteoliposomes at physiological concentrations, inhibited the transport activity; differently, the cations had no effect when included in the internal proteoliposome compartment. This data highlighted an asymmetric regulation of *Sl*CAT2. Cholesteryl hemisuccinate, included in the proteoliposomal membrane, stimulated the *Sl*CAT2 transport activity. The homology model of the protein was built using, as a template, the 3D structure of the amino acid transporter *Gk*ApcT. Putative substrate binding residues and cholesterol binding domains were proposed. Altogether, the described results open new perspectives for studying the response of *Sl*CAT2 and, in general, of plant vacuolar transporters to metabolic and environmental changes.

## 1. Introduction

The vacuole is the largest organelle in plant cells and it plays several roles. Originally, this organelle was considered mainly responsible for cell turgor; then, it became clear that the vacuole is involved in protein digestions, storage of water, ions, and metabolites as well as toxic compounds [1,2]. Recently, the vacuole received more attention because is involved in plant metabolism, pH homeostasis, stress responses, cell growth and development [2,3,4], and signal transduction [3,5]. In this frame, many proteins are expected to accomplish these functions [1,3]. In fact, the vacuolar membrane hosts different transporters with specificity towards several classes of molecules. Traffic of amino acids across the vacuolar membrane is crucial for plant cell homeostasis [1,2,6]. However, the characterization of transporters in the vacuolar membrane is not straightforward due to the inaccessibility of these transporters from the extracellular environment. To overcome this problem, the vacuolar transporter CAT2 from *S. lycopersicum* (*Sl*CAT2) has been recently characterized in the in vitro system of proteoliposomes obtained by reconstituting the recombinant protein over-expressed in *E. coli* [7].

In this experimental model, it was revealed that *Sl*CAT2 is involved in the transport of the cationic amino acids arginine and lysine and of the nonproteogenic amino acid ornithine. The transport is regulated by ATP, which probably binds to an N-terminus domain of the protein [7]. Besides the mentioned amino acids, acetylcholine is also a substrate of *Sl*CAT2. Interestingly, this function might be related to the non-neuronal cholinergic system present also in plants and involved in the regulation of cell elongation, water homeostasis, and photosynthesis [8]. In-line with this, *Sl*CAT2 belongs to the APC family (amino acid polyamine choline) which includes transporters conserved in all living organisms, i.e., from bacteria to humans [9,10,11].

Despite the importance of membrane transporters for vacuolar homeostasis, the studies of regulatory aspects of these proteins are still at their infancy. One of the factors involved in maintaining the vacuolar homeostasis is the concentration of some cations [12]. In this frame, the V-ATPase, the V-PPase, the aquaporin channel, and the ion exchangers are the most known membrane components, which regulate the intravacuolar concentrations of cations including H^+^. The V-ATPase accumulates H^+^ inside the vacuolar lumen upon cytosolic ATP hydrolysis with a complex molecular mechanism resembling that of F-type ATPase of mitochondria and chloroplasts [13,14,15,16]. The V-PPase is also involved in H^+^ uptake into the vacuole but uses the energy deriving from PPi hydrolysis in the cytosol [13,14,17,18]. The constant activity of these proteins allows the maintenance of a ΔpH, that can reach two to three units, and a ΔΨ of 30 mV positive inside [13,14]. The aquaporins, referred to as TIPs (tonoplast intrinsic proteins), are responsible for the traffic of water and other small molecules in vacuole to balance the osmotic pressure and to respond to salt stress [14,19,20]. Na^+^ is the main player in salt stress regulation; its concentration in cytosol normally ranges from 20 to 50 mM. Under conditions of high salinity, the vacuolar concentration of Na^+^ can exceed 10 times the cytosolic one [21,22]. K^+^ represents one of the most important nutrients for cell growth and development being the cofactor of several enzymes [1,21,23,24]. It is also involved in turgor-driven processes such as stomatal movements and cell growth by distention [13,25]. For these reasons, the vacuolar concentration of K^+^ can vary from 20 to 200 mM, while the cytosolic concentration is kept quite constant, roughly 100 mM [22,26].

Nitrogen is a limiting factor for plant growth and development [13]. The major source of nitrogen is represented by ammonia (NH_4_^+^/NH_3_) and urea. The equilibrium between NH_4_^+^ and NH_3_ depends on the cytosolic pH. Since the vacuolar pH is more acidic than the cytosolic one, NH_4_^+^ is the prevailing vacuolar form and its concentration in vacuole is 100-fold higher than in cytosol [13].

Among divalent cations, Mg^2+^ and Ca^2+^ are the most abundant in plant cells. Mg^2+^ is involved in several processes such as conformational stabilization of macromolecules, chlorophyll synthesis, enzyme activation, and osmotic regulation together with K^+^. In-line with these regulatory features, a high level of Mg^2+^ or its deficit in the soil could be destructive for plant life [27]. Therefore, vacuoles play an essential role also in the Mg^2+^ homeostasis, being able to accumulate Mg^2+^ up 80 mM [28], while in the cytosol the Mg^2+^ concentration is approximately 0.2–0.4 mM [1]. 

As in animal cells, Ca^2+^ plays the role of second messenger in plants and is responsible for the activation of several signaling pathways. As an example, Ca^2+^ is involved in stomatal movements: stomatal closure is linked to abscisic acid stimulation caused by the opening of vacuolar Ca^2+^ channels with the consequent increase in cytosolic Ca^2+^ concentration [29]. Ca^2+^ is also important to neutralize vacuolar anions to strengthen cell walls for responding to stress [30]. 

The cytosolic Ca^2+^ concentration is ~200 nM, while the vacuolar concentration is three orders of magnitude higher. Therefore, an energy-dependent process is required to accumulate Ca^2+^ within the vacuole. Indeed, a primary energized Ca^2+^-ATPase and a secondary energized Ca^2+^ exchanger mediate the process [13,29,31].

From the depicted scenario, it is evident that the described ion distribution may play some role in the regulation of transporters working in the vacuolar membrane [32]. 

Therefore, the present study sought to define some regulatory properties of *Sl*CAT2, among which the response to cations. In order to correlate the role of this protein to tomato physiology, the experiments have been performed taking into consideration the physiological composition of intra- and extraluminal milieu. Indeed, tomato crop is very relevant for both nutrition and biotechnology purposes and, therefore, the knowledge of tomato biology is an up-to-date field of investigation.

## 2. Results

### 2.1. Orientation of the SlCAT2 Reconstituted in Proteoliposomes

**Figure 1:**
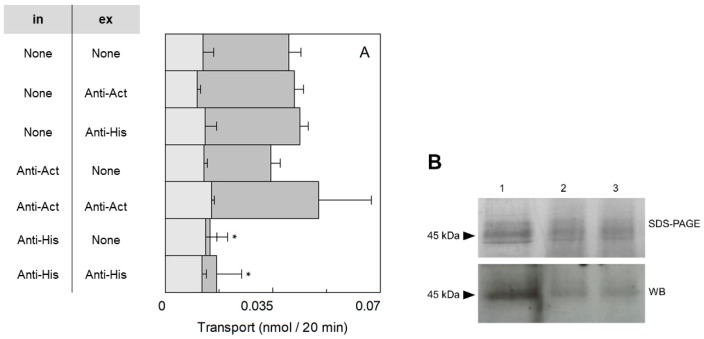
Sidedness of the reconstituted *Solanum lycopersicum* CAT2 (*Sl*CAT2) in proteoliposomes. (**A**) *Sl*CAT2 was purified and reconstituted in proteoliposomes as described in Section 4.4. The purified protein was preincubated or not, for 30 min under rotatory shaker at room temperature, with 6 µg of anti-His or anti-Actin antibody, as indicated in the table (in; intraliposomal compartment). Transport was measured in 20 min, as described in Section 4.5, by adding 100 μM [^3^H]Arg to proteoliposomes containing 200 mM sucrose and 15 mM ATP at pH 7.5. In the external compartment, 0.6 µg of anti-His or anti-Actin were added or not, as indicated in the table (ex; extraliposomal compartment). The transport in proteoliposomes (dark gray bars) and in liposomes (light gray bars) is measured as nmol of radioactive substrate taken up in 20 min. Results are the means ± SD from three experiments. (*), significantly different from the control (none) for *p* < 0.05 as calculated from 1-way ANOVA test. (**B**) Sodium dodecyl sulfate polyacrylamide gel electrophoresis (SDS-PAGE) of 0.15 µg purified *Sl*CAT2 (lane 1) and the corresponding protein volume of *Sl*CAT2 reconstituted in proteoliposomes not preincubated with antibody (lane 2), after preincubation with anti-His antibody (lane 3). Proteoliposomes were purified by size-exclusion chromatography as described in Section 4.5 prior to SDS-PAGE run.

To assess the orientation of the *Sl*CAT2 transporter in the proteoliposomal membrane, a method based on side-specific targeting was employed [33]. In particular, an antibody against the 6His-tag has been used exploiting the location of the 6His-tag at the C-terminus of the recombinant protein. The interaction between the antibody and the 6-His tag of the protein should create a steric hindrance impairing the transport activity, as previously observed in the case of other transporters [33,34]. After the reconstitution procedure, the C-terminus portion of the protein may be exposed towards the internal or the external side of the proteoliposomes. To discriminate between the two possibilities, *Sl*CAT2 was incubated with the anti-His antibody before or after the insertion into the proteoliposomal membrane. In the first case, the antibody can reach both sides (internal or external) of the recombinant protein. In the second case, the antibody can bind only to the protein side exposed towards the extraliposomal compartment. After incubating the anti-His in the two different conditions, the transport activity has been measured as [^3^H]Arg uptake in proteoliposomes [7]. Transport inhibition was observed upon incubation of the antibody with the protein before the insertion in the membrane, while no significant effect could be observed upon addition of the antibody to the external side (Figure 1A). The addition of anti-His antibody to the external side of proteoliposomes prepared with protein pretreated with anti-His antibody did not further increase the inhibitory effect (Figure 1A). In parallel samples, an anti-actin antibody was added to the protein before reconstitution, as control; in this case, no effect was observed indicating that, indeed, the inhibition was specifically due to the targeting of the 6His-tag sequence located at the C-terminus of the recombinant *Sl*CAT2. The same experiment was performed using liposomes, i.e., without reconstituted protein, and no effect by addition of any antibody was measured (light gray bar in Figure 1A). It has to be stressed that the [^3^H]Arg accumulated in the control liposomes (with no reconstituted protein) was always less than 30% with respect to that taken up by proteoliposomes. This data clearly indicates that the 6His-tag is exposed towards the internal side of the protein inserted in the proteoliposomal membrane. The actual presence of *Sl*CAT2 in the membrane was tested by separating proteoliposomes deriving from experiment in Figure 1A by size exclusion chromatography and applying the samples on SDS-PAGE and WB analysis after extraction by detergent (Figure 1B). As shown by the figure, the reconstituted protein was ~30% with respect to the protein added to the reconstitution mixture in all the analyzed conditions.

### 2.2. Regulation of the SlCAT2 Transport Activity by pH and Osmolality

**Figure 2:**
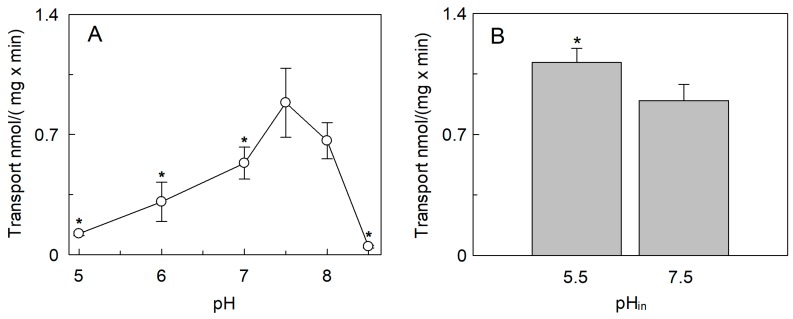
Effect of pH on the transport activity of SlCAT2 in proteoliposomes. *Sl*CAT2 was purified and reconstituted in proteoliposomes as described in Section 4.4. (**A**) Transport rate (nmol/(mg × min)) was measured in 10 min, as described in Section 4.5, by adding 100 μM [^3^H]Arg to proteoliposomes containing 200 mM sucrose and 15 mM ATP. The pH was kept equal in both the internal and external site of proteoliposomes. (**B**) To generate the pH gradient, transport was measured in 10 min, as described in Section 4.5, by adding 100 μM [^3^H]Arg (pH 7.5) to proteoliposomes prepared using the buffer at pH 7.5 or pH 5.5, as indicated (pH_in_) and containing 200 mM sucrose and 15 mM ATP. Results are the means ± SD from three experiments. (*), Significantly different, from the control at pH_in_ 7.5, for *p* < 0.05 as calculated from the Student’s *t*-test analysis.

The activities of the plant transporters *At*CAT1, *At*CAT5, and *At*CAT6 which share 26.8%, 25%, and 26.3% of identity with *Sl*CAT2, respectively, are dependent on pH [9]. Therefore, the transport activity of *Sl*CAT2 was evaluated at a pH ranging from pH 5.0 to pH 8.5. As shown in Figure 2A, the pH dependence of the [^3^H]Arg uptake showed a bell-shaped behavior with an optimum at pH 7.5. The effect of a pH gradient artificially created at the two sides of the proteoliposomes was also tested. A small but significant increase of transport activity was found in the presence of a pH gradient acidic inside (pH 5.5_in_/pH7.5_out_) with respect to the condition of equal internal and external pH 7.5 (Figure 2B).

**Figure 3:**
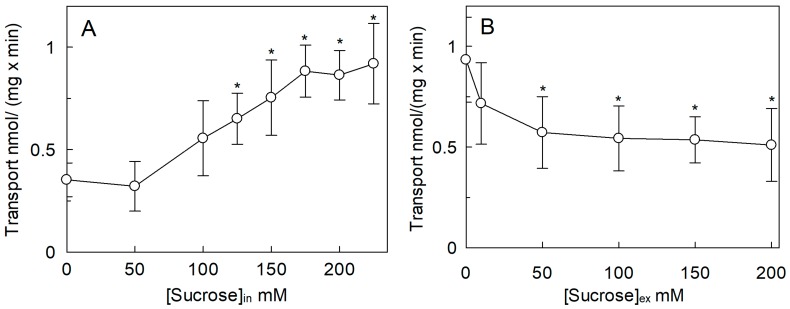
Effect of osmotic pressure on the transport activity of *Sl*CAT2 in proteoliposomes. *Sl*CAT2 was purified and reconstituted in proteoliposomes as described in Section 4.4. (**A**) Transport rate (nmol/(mg × min)) was measured in 10 min, as described in Section 4.5, by adding 100 μM [^3^H]Arg to proteoliposomes containing indicated concentrations of sucrose and 15 mM ATP, at pH 7.5. (**B**) Transport rate was measured in 10 min, as described in Section 4.5, by adding 100 μM [^3^H]Arg together with indicated concentrations of sucrose to proteoliposomes containing 200 mM sucrose and 15 mM ATP, at pH 7.5. Results are the means ± SD from three experiments. (*), Significantly different from the control (without sucrose in both (**A**) and (**B**)) for *p* < 0.05 as calculated from Student’s *t*-test analysis.

To evaluate also the possible influence of osmolality on the activity of *Sl*CAT2, sucrose was used as an osmolyte. The effect was studied by changing the concentration of sucrose in the intraliposomal compartment (Figure 3A). In this condition, the [^3^H]Arg uptake increased approximately three times when the intraliposomal osmolality was in the range of 50 to 175 mOsmol, reaching a plateau up to 225 mOsmol. To evaluate the side-specificity of such a regulation, the same experiment was also conducted as dependence on the external osmolality (Figure 3B). Differently from the internal hyperosmolality, the increase in external osmolality caused a reduction of [^3^H]Arg uptake to about 45% of the control at 50 mM sucrose, i.e., 0.51 ± 0.18 with respect to the control 0.93 ± 0.2 nmol/(mg × min); this reduction did not change by further increasing the external osmolality.

### 2.3. Effect of Cations on the SlCAT2 Transport Activity

**Figure 4:**
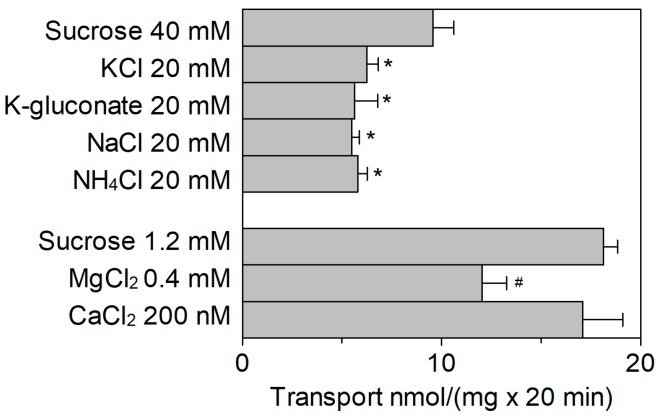
Effect of extraliposomal cations on the transport activity of *Sl*CAT2. *Sl*CAT2 was purified and reconstituted in proteoliposomes as described in Section 4.4. Transport was measured in 20 min, as described in Section 4.5, by adding 100 μM [^3^H]Arg together with the indicated compounds to proteoliposomes containing 200 mM sucrose and 15 mM ATP, at pH 7.5. Concentrations were chosen according to the average concentrations of cations in the cytosol. Results are the means ± SD from four experiments. (*), significantly different from the control (sucrose 40 mM) for *p* < 0.05 as calculated from 1Way ANOVA test. (#), significantly different from the control (sucrose 1.2 mM) for *p* < 0.05 as calculated from 1-way ANOVA test.

**Figure 5:**
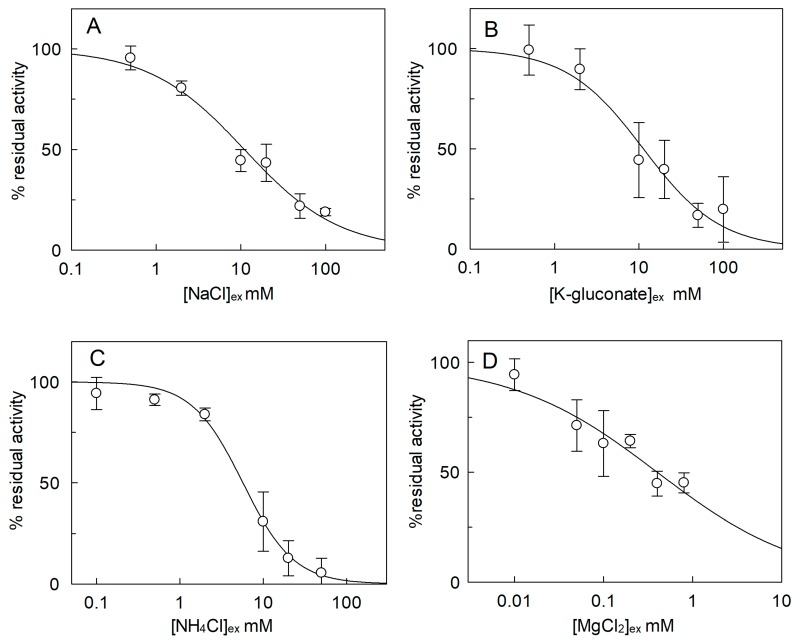
Dose–response curve for the inhibition of *Sl*CAT2 by external cations. *Sl*CAT2 was purified and reconstituted in proteoliposomes as described in Section 4.4. Transport was measured in 20 min, as described in Section 4.5, by adding 100 μM [^3^H]Arg together with the indicated concentrations of NaCl (**A**), K-gluconate (**B**), NH_4_Cl (**C**), or MgCl_2_ (**D**) to proteoliposomes containing 200 mM sucrose and 15 mM ATP, at pH 7.5. Transport activity was calculated as the percent of residual activity with respect to condition without any addition (in absence of indicated compounds). Results are the means ± SD from three experiments.

The [^3^H]Arg uptake was measured in the presence of KCl, NaCl, NH_4_Cl, CaCl_2_, or MgCl_2_ added to the external proteoliposome compartment (Figure 4). The cation concentrations were kept close to their physiological concentrations in cytosol [21,22,24,26,28,29]. The [^3^H]Arg uptake was inhibited by external KCl. The effect of KCl was compared to that of K-gluconate to discriminate the possible influence by Cl^−^ from that of K^+^; no difference between the two K^+^ salts was observed indicating that the inhibition was specifically due to K^+^. Na^+^ and NH_4_^+^ caused an inhibition similar to that of K^+^, i.e., ~40% with respect to 40 mM sucrose used as the control of osmolality. Notably, sucrose per se, when used at 40 mM, caused a reduction of transport activity with respect to the condition without sucrose, in-line with the results of Figure 3B. Among divalent cations, Mg^2+^ exerted an inhibition of 33%, while Ca^2+^ did not exert a significant effect with respect to sucrose used as control of osmolality. It has to be stressed that no difference of activity was observed in the presence of 1.2 mM sucrose with respect to control without sucrose (see Figure 3B). Dose–response analyses were performed for those cations, which exerted a stronger inhibition on the extraliposomal (cytosolic) side of the protein (Figure 5). From these experiments an IC_50_ value of 10.9 ± 0.8 mM, 11.3 ± 1.2 mM, 5.7 ± 1.49 mM, or 0.42 ± 0.18 mM was derived for Na^+^ (Figure 5A), K^+^ (Figure 5B), NH_4_^+^ (Figure 5C), or Mg^2+^ (Figure 5D), respectively.

**Figure 6:**
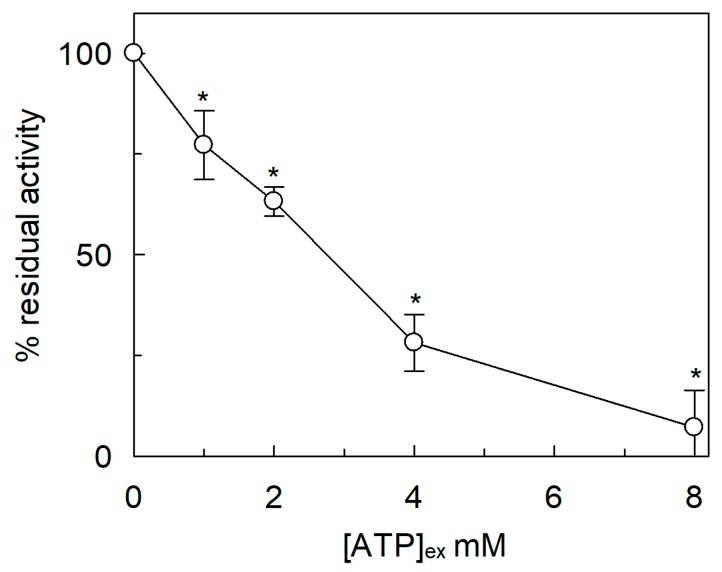
Effect of ATP(Na^+^)_2_ salt on the transport activity of *Sl*CAT2 in proteoliposomes. *Sl*CAT2 was purified and reconstituted in proteoliposomes as described in Section 4.4. Transport was measured in 20 min, as described in Section 4.5, by adding 100 μM [^3^H]Arg together with the indicated concentrations of ATP to proteoliposomes containing 200 mM sucrose and 15 mM ATP, at pH 7.5. Transport activity was calculated as the percent of residual activity with respect to condition without any addition (in absence of external ATP). Results are the means ± SD from three experiments. (*), Significantly different, from the control (without ATP in the extraliposomal compartment) for *p* < 0.05 as calculated from Student’s *t*-test analysis.

The sidedness of *Sl*CAT2 transport was further investigated by testing the effect of ATP on the extraliposomal side (Figure 6). Differently from the internal side, ATP inhibited *Sl*CAT2-mediated [^3^H]Arg uptake when used at concentrations within the same range of those activating the transport from the intraliposomal compartment [7], indicating that the activation by ATP is side-specific. However, the effect of ATP, used as (Na^+^)_2_ salt, is partially due to the inhibition by Na^+^ (Figure 5A) and osmolality (Figure 3B). Since the cytosolic concentration of free-ATP is in the micromolar range [35] the observed inhibition might not be physiologically relevant.

**Figure 7:**
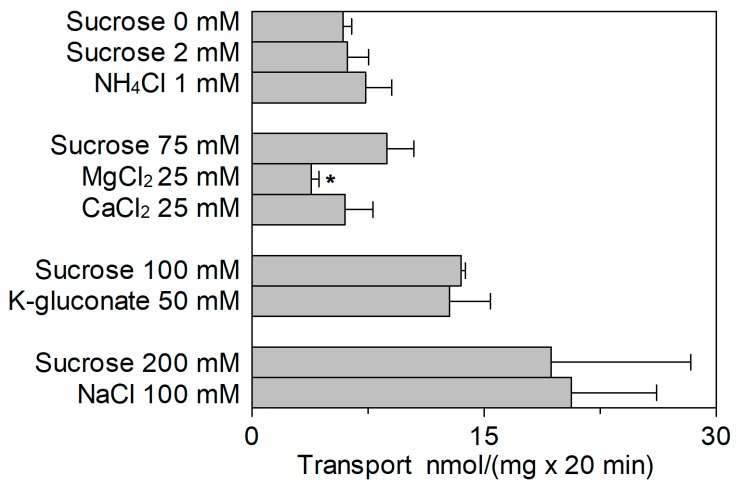
Effect of intraliposomal cations on the transport activity of *Sl*CAT2. *Sl*CAT2 was purified and reconstituted in proteoliposomes as described in Section 4.4. Transport was measured in 20 min, as described in Section 4.5, by adding 100 μM [^3^H]Arg to proteoliposomes containing indicated concentrations of sucrose or cations and 15 mM ATP, at pH 7.5. Concentrations were chosen according to the average concentrations of cations in the vacuolar lumen. Results are the means ± SD from four experiments. (*), Significantly different from the control (isoosmotic concentration of sucrose for each category of cations) for *p* < 0.05 as calculated from 1-way ANOVA test.

**Figure 8:**
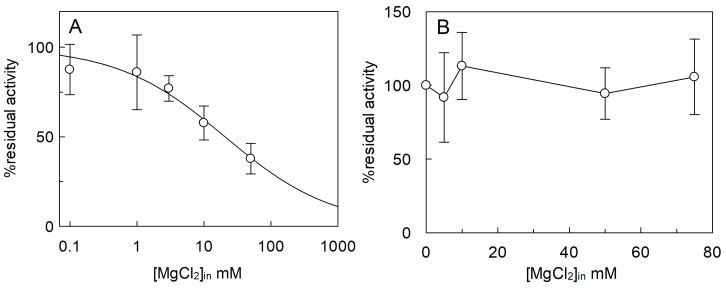
Effect of intraliposomal Mg^2+^ on the transport activity of *Sl*CAT2. *Sl*CAT2 was purified and reconstituted in proteoliposomes as described in Section 4.4. (**A**) Dose–response curve for the inhibition of Mg^2+^ in the presence of intraliposomal ATP. Transport was measured in 20 min, as described in Section 4.5, by adding 100 μM [^3^H]Arg to proteoliposomes containing the indicated concentrations of Mg^2+^, 200 mM sucrose, and 15 mM ATP at pH 7.5. (**B**) Analysis of the inhibition of Mg^2+^ in the absence of intraliposomal ATP. Transport was measured in 20 min, as described in Section 4.5, by adding 100 μM [^3^H]Arg to proteoliposomes containing the indicated concentrations of Mg^2+^ and 200 mM sucrose. Transport activity was calculated as the percent of residual activity with respect to condition without any addition (in absence of intraliposomal Mg^2+^). Results are the means ± SD from three experiments.

**Figure 9:**
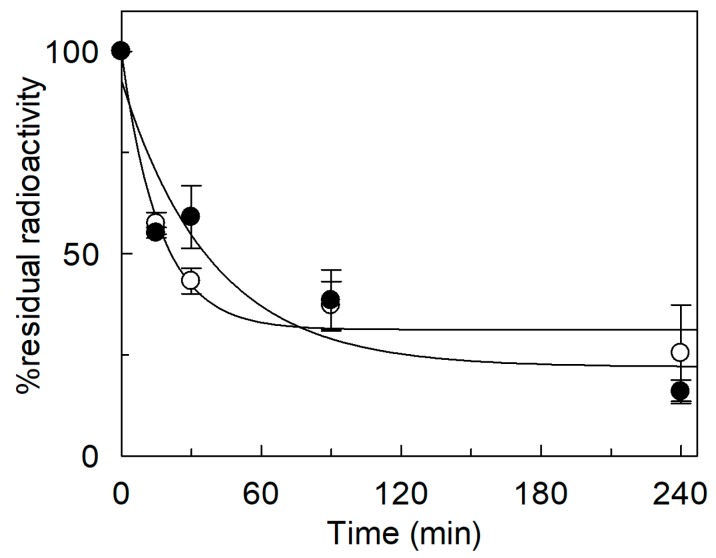
Efflux from proteoliposomes reconstituted with *Sl*CAT2. *Sl*CAT2 was purified and reconstituted in proteoliposomes as described in Section 4.4. Arginine efflux was measured at the indicated time, as described in Section 4.5. Internal residual radioactivity was measured as percent with respect to control (time 0), in the absence (open circle) or in the presence (closed circle) of 0.45 mM Mg^2+^ in the extraliposomal compartment. Data are plotted using the single exponential equation with offset as described in Section 4.5. Results are the means ± SD from three experiments.

The effect of cations in the internal compartment was also evaluated (Figure 7). In this case, activation of the transporter by both K^+^ and Na^+^ was observed, with respect to the condition without osmolytes. The extent of activation, however, corresponded to that of the isoosmolar concentration of sucrose (Figure 7), indicating that the activation was due to an osmotic effect (see also Figure 3A). Ca^2+^ and NH_4_^+^ did not exert any significant effect as in the case of K^+^ and Na^+^. On the contrary, Mg^2+^ had a strong inhibitory effect. Therefore, a dose–response analysis of the Mg^2+^ inhibition from the internal side was performed (Figure 8A) and an IC_50_ value of 23.0 ± 9.9 mM was derived, which is two orders of magnitude higher than that measured on the external side (Figure 5D). As previously described, *Sl*CAT2 activity is stimulated by intraliposomal ATP [7], that is present inside the proteoliposomes in all the experiments. Therefore, the effect of Mg^2+^ could be due to the known interaction with ATP. In order to dissect the effect of the sole Mg^2+^, transport was also assayed in the absence of internal ATP (Figure 8B). The inhibition of *Sl*CAT2 by Mg^2+^ virtually disappeared when ATP was omitted, confirming the above mentioned hypothesis (Figure 8B). This result definitively demonstrated that Mg^2+^, as the other cations, does not exert any direct effect on *Sl*CAT2 from the internal (luminal) side. For the sake of clarity, it has to be stressed that in absence of ATP, the transport activity of *Sl*CAT2 is much lower than that measured in the presence of ATP, explaining the higher scattering observed in the transport measurements (Figure 8B) across different experiments. *Sl*CAT2 catalyzes arginine efflux as well [7]. Then, the effect of Mg^2+^ on the external side was also tested on the [^3^H]Arg efflux measured from preloaded proteoliposomes in the presence or absence of external Mg^2+^. Interestingly, the presence of the cation in the external compartment had no, or only a slight, effect on the [^3^H]Arg efflux (Figure 9).

### 2.4. Effect of Cholesterol on the SlCAT2 Transport Activity

**Figure 10:**
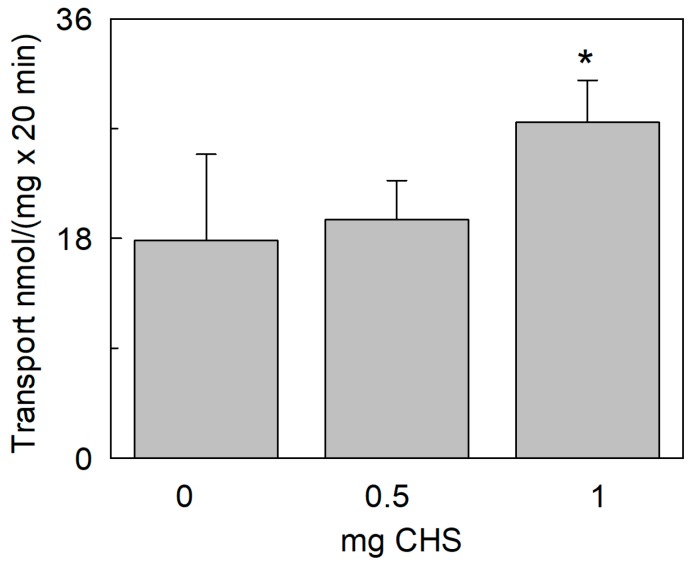
Effect of cholesteryl hemisuccinate (CHS) on the transport activity of *Sl*CAT2. *Sl*CAT2 was purified and reconstituted in proteoliposomes as described in Section 4.4. Transport was measured in 20 min, as described in Section 4.5, by adding 100 μM [^3^H]Arg to proteoliposomes containing 200 mM sucrose and 15 mM ATP at pH 7.5. Proteoliposomes are prepared without CHS or with 0.5 mg or 1 mg CHS in 1 mL of proteoliposomes as described in Section 4.6. Results are the means ± SD from four experiments. (*), significantly different from the control (condition with no addition of CHS) for *p* < 0.05 as calculated from Student’s *t*-test analysis.

The vacuole membrane contains 30% sterols [36]. Since cholesterol was found to be important in the interaction and in the stabilization of several membrane proteins, we have exploited the suitability of the proteoliposome model in changing the lipid composition. Cholesteryl hemisuccinate (CHS), a commercial form of cholesterol with a relatively higher water solubility than cholesterol and widely used in this kind of applications [37,38,39,40], was included in the proteoliposomal membrane. The transport activity of *Sl*CAT2 was measured in the presence of two different amounts of CHS. Interestingly, the [^3^H]Arg uptake was stimulated by increasing amounts of CHS up to 30% in the presence of 1 mg of CHS corresponding to 10% of total lipid composition (Figure 10).

### 2.5. Homology Model

**Figure 11:**
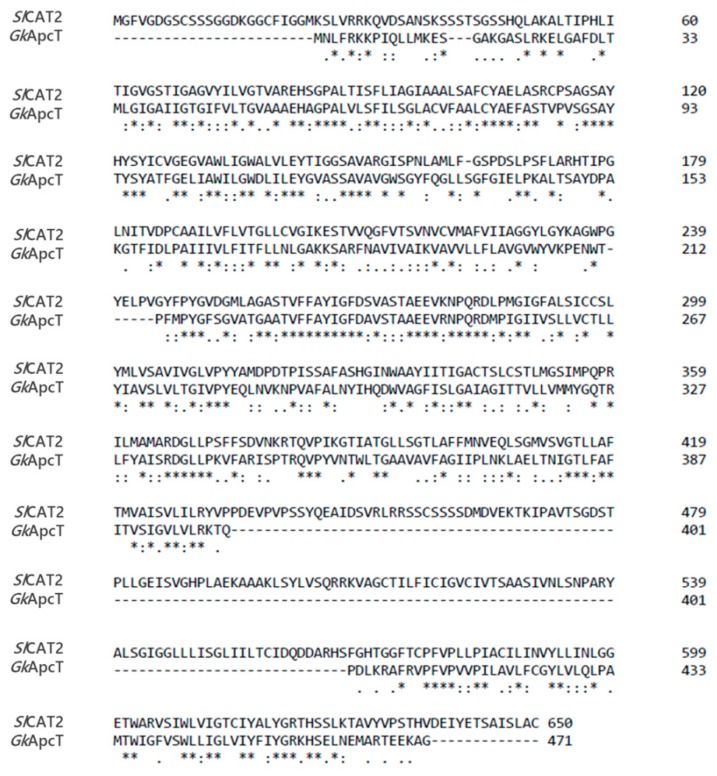
The sequences of *Sl*CAT2 and *Gk*ApcT were aligned using the software Clustal Omega as described in Section 4.7.

**Figure 12:**
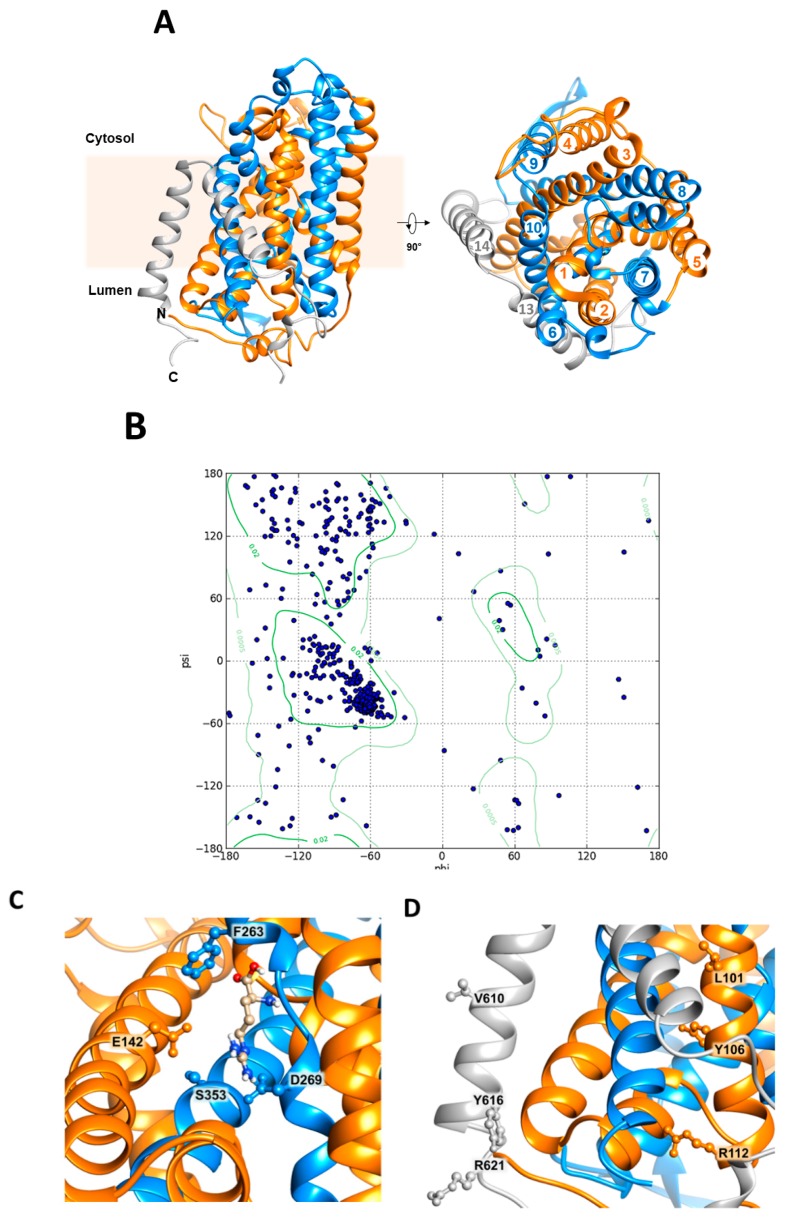
Homology structural model of *Sl*CAT2. The three-dimensional model is built, as described in Section 4.7, using the *Gk*ApcT atomic coordinates (5OQT) as a template. The α-helices are colored in orange or in blue to identify the pseudo two-fold symmetry axis of LeuT-fold. The α-helices which are not part of LeuT fold are colored in gray. The model is designed using the Chimera 1.13.1 software. (**A**) The protein is inserted in the membrane with the N- and C-termini in the luminal space (left-front view); on the right, the model is rotated by 90 °C showing the top view and α-helices are numbered. (**B**) Ramachandran plot of the model in A with residues in favored (smaller gate) and allowed (larger gate) regions. The residues are depicted as blue circles. (**C**) Molecular docking of arginine in the putative binding site of *Sl*CAT2; residues putatively responsible for arginine binding and gating (S353, D269, and F263) together with the residue putatively responsible for proton coordination (E142) are indicated in the ball and stick representation. (**D**) Residues of CRAC motifs responsible for cholesterol binding are highlighted in TM 2 (orange α-helix) and TM 14 (gray α-helix) in the ball and stick representation; numbering of α-helices corresponds to that of Figure 12A.

As previously reported, *Sl*CAT2 has 14 putative transmembrane segments and it should be oriented, in the vacuolar membrane, with N- and C-termini exposed towards the internal side [7,9] in agreement with the data shown in Figure 1. The three-dimensional structure of any CAT member is not solved, yet. The transporter shares 27% identity (Figure 11) with the prokaryotic transporter *Gk*ApcT (*G. kaustophilus*) responsible for proton-coupled amino acid transport across the plasma membrane [41]. Therefore, to gain some information on *Sl*CAT2 fold, the homology model was constructed on the basis of the crystal structure of *Gk*ApcT (Figure 12A). As shown by the Ramachandran plot (Figure 12B), the obtained structure was suitable, although it lacks two transmembrane domains (TM 11 and TM 12). In particular, 84.8% or 93.9% of all residues were in favored or in allowed regions, respectively, as derived by MolProbity software [42]. In this structure, it was possible to identify the typical APC superfamily fold, with transmembrane α-helices TM1-TM5 (orange) linked to α-helices TM6-TM10 (blue) by a pseudo-two-fold symmetry axis (Figure 12A). The binding of arginine could be predicted on the basis of the position of the amino acid substrate in *Gk*ApcT. Phe263 (Figure 12C) is homologous of Phe231 that is responsible for substrate gating in *Gk*ApcT. Interestingly, this residue is also conserved in the human histidine transporter LAT1 (Phe 252) which belongs to the same APC superfamily [43].

## 3. Discussion

The majority of the studies on plant transporters has been conducted so far, on *A. thaliana*, whose genome has been sequenced revealing the existence of 14 APC genes. However, studying *S. lycopersicum* transporters might be in some instances, even more interesting due to the applications of tomato in biotechnology. Arginine is the highest affinity substrate of *Sl*CAT2, as previously shown using the recombinant protein reconstituted into proteoliposomes [7]. Noteworthy, a relatively high concentration of arginine into the vacuole has been measured [44]. The importance of this amino acid for plants mainly resides in its high nitrogen content and in its involvement in polyamines and nitric oxide (NO) synthesis, which is important during fruit development [44]. 

On the basis of the data here presented, the *Sl*CAT2 inserts asymmetrically into the proteoliposomal membrane (Figure 1 and Figure 12) allowing both arginine uptake and efflux. The asymmetric insertion is further confirmed by the side-specific regulation observed for both osmolyte and cations, which exert opposite effects at the two sides of the membrane. The side-specific regulation of *Sl*CAT2 suggests that the protein is inserted in the proteoliposomal membrane with an orientation that may correspond to that in the native membrane of vacuoles (right-side-out). This is consistent with the detergent removal reconstitution procedure that forms proteoliposomes with a relatively small radius. This physical property forces the orientation of the membrane proteins as shown for several transporters from either plasma membrane or intracellular organelles [45,46,47,48,49]. Notably, the percentage of reconstituted *Sl*CAT2 is ~30%, in-line with previous literature data demonstrating that the average of reconstitution efficiency is in the range of 10 to 30% [50,51]. We cannot absolutely exclude that the orientation of the transporter in proteoliposomes could be the opposite of the native membrane, even though this would not fit with the data on regulation. In fact, it is well known that the intravacuolar osmolality can undergo wide oscillations in normal conditions. In good agreement with this physiological phenomenon, also the activity of the *Sl*CAT2 is modulated only by the increase of intraliposomal, i.e., intravacuolar, osmolality. This may ensure a fast response of the transporter to osmotic changes in vivo [52,53,54]. On the contrary, increased external (cytosolic) osmolality has an opposite effect with a slight inhibition occurring in conditions of high osmotic imbalance, which may not be physiological [54]. 

The side-specific effect of ions is in favor of a right-side-out orientation, too. In fact, in the external compartment, i.e., cytosolic, besides a plausible sucrose-similar osmotic effect, an additional effect by cations, superimpose the osmotic one. On the contrary, in the intraliposomal side, i.e., vacuolar lumen, the effects of ions are negligible. Noteworthy, the response of the protein to ions is coherent with their cytosolic/luminal concentrations being in favor of a largely right-side-out orientation of the protein. In the frame of regulation by cations, Mg^2+^ revealed to be particularly interesting: this divalent cation does not exert direct effects on the transporter when present in the internal side. The apparent inhibition observed is due to the formation of an Mg-ATP complex which reduces the concentration of free ATP, necessary for *Sl*CAT2 full activity [7]. At this stage, we cannot assess with certainty whether or not the ATP sequestering effect in the vacuolar lumen, may be physiologically relevant. Differently from the luminal side, Mg^2+^ inhibits the transporter from the cytosolic (extraliposomal) side even at low concentration. However, this effect is observed on the arginine uptake while there is no transinhibition on the arginine efflux. Therefore, it can be hypothesized that the regulation by Mg^2+^ affects arginine storage, not its release from vacuoles. The asymmetric effect of Mg^2+^ further supports the side-specific (right-side-out) orientation of the transporter. Possible physiological regulators could induce arginine remobilization from the vacuole under specific physiopathological conditions. Some clues can also derive from bioinformatics by analyzing the 3D homology model built in the present work (Figure 12). As an example, Glu115 involved in proton binding on the internal side in *Gk*ApcT may suggest that the corresponding homologous Glu142 of *Sl*CAT2 might be involved in the binding of a proton in *Sl*CAT2, as well (Figure 12C). At this stage, it cannot be ascertained if a proton moves across the membrane or only binds to a regulatory site. Counter-translocation of a proton may compensate for the movement of the positive charge of arginine. Alternatively, a proton might only bind and be released from the protein allowing some conformational changes which facilitate arginine transport as shown for other transporters [55]. The physical interaction between the transporter *Gk*ApcT and cholesterol has been proposed to mediate the connection of the transporter with another bacterial protein with a single membrane-spanning domain [41]; interestingly, the molecule of cholesterol could interact with *Sl*CAT2 at transmembrane domains 2 and 14. In this region, in fact, we identified two possible cholesterol binding sites on the basis of the algorithm of CRAC motif (L/V)-X_1–5_-(Y)-X_1–5_-(K/R) (Figure 12D) [41,56]. However, additional site-directed mutagenesis and structural and topological studies are required to deal with these aspects, including the definitive assessment of the orientation of the transporter in the artificial and native membranes. Finally, the function of arginine uptake or efflux and its diverse modulation may be linked also to a more complex phenomenon, i.e., intracellular amino acid sensing, whose study in the plant is yet underdeveloped, as recently pointed out [32]. Amino acid sensing is, in fact, a well-described phenomenon in animals and yeast where availability of these molecules is responsible for modulation of mTOR and GCN2 pathways involved in cell growth, development, and metabolism regulation [57]. In particular, amino acid transporters of the plasma membrane as well as of intracellular organelles may be considered a “transceptor”, i.e., low capacity transporter with a receptor function able to transmit the availability of a specific nutrient for sensing machinery [48,57]. In this scenario, arginine is one of the amino acids known to play a role in the mentioned signaling function in cells via mTOR pathway modulation [58]. Therefore, the results here presented suggest that *Sl*CAT2 may be also involved in sensing pathways via mobilization of arginine from vacuoles.

In conclusion, the proteoliposome experimental model allowed us to characterize the transport mediated by *Sl*CAT2. Some information on osmotic as well as salt regulation of *Sl*CAT2 were gained by exploiting the suitability of proteoliposomes in handling and precisely manipulating the experimental conditions in the internal and external compartments. Even though the experiments were conducted exquisitely in vitro, this work highlights one of the few examples of the influence of cations and osmolytes at physiological concentrations on the activity of a plant transporter. To uncover the structural basis of these regulatory responses, future work using bioinformatics coupled to site-directed mutagenesis will be carried out.

## 4. Materials and Methods

### 4.1. Materials

*E. coli* Rosetta(DE3)pLysS cells were from Novagen (Rome, Italy); ECL plus, Hybond ECL membranes were from GE Healthcare; l-[^3^H]Arg was from Perkin Elmer (Waltham, MA, USA); conjugated anti-His6 antibody, TX-100, Amberlite XAD-4, egg yolk phospholipids (3-sn-phosphatidylcholine from egg yolk), Sephadex G-75, His-Select resin, l-Arg and all the other reagents were from Sigma-Aldrich (Saint Louis, MO, USA).

### 4.2. Protein Production

Heterologous expression of SlCAT2 E. coli Rosetta(DE3)pLysS cells (Novagen) were transformed with pET21-SlCAT2 (pET21 from Novagen and SlCAT2 amplified and cloned in Indiveri’s lab) as previously described [7]. In brief, *E. coli* Rosetta(DE3)pLysS cells carrying the pET21-SlCAT2-6His were preinoculated in LB medium, supplemented with same concentrations of ampicillin and chloramphenicol, and grown overnight at 37 °C under rotatory stirring (300 rpm). After overnight growth, the saturated inoculum was diluted 1:10 in the same LB medium supplemented with the same concentrations of ampicillin and chloramphenicol. Then, at an OD of ~0.8 measured at 600 nm, 0.4 mM isopropyl-β-d-thiogalactopyranoside (IPTG) was added to induce protein synthesis. After 4 h of growth at a temperature of 28 °C, cells were harvested by centrifugation at 3000× *g* for 15 min at 4 °C. The bacterial pellets were resuspended in a buffer containing 20 mM Hepes Tris pH 7.5 and 300 mM NaCl supplemented with protease inhibitor cocktail. Thus, cells were lysate by mild sonication at 4 °C (10 min in pulses of 1 s sonication, 1 s intermission) with a Vibracell VCX-130 sonifier (SONICS). The soluble and the insoluble fractions were separated by centrifugation at 12,000× *g* for 5 min at 4 °C. The proteins of the cell lysates were analyzed by 12% SDS-PAGE. 

### 4.3. Protein Purification

The protein was purified as previously described [7] with some modifications: in brief, the insoluble fraction was treated with 10 mM DTE, 3.2 M urea, 0.8% Sarkosyl, 200 mM NaCl, 20 mM Tris, and HCl pH 8.0 and centrifuged at 12,000× *g* for 10 min at 4 °C. One milliliter of the solubilized lysate was applied onto a column filled with His-select Ni-Chelating affinity gel (0.5 cm diameter, 3 cm height) preconditioned with 8 mL of a buffer containing 0.1% Sarkosyl, 200 mM NaCl, 10% glycerol, and 20 mM Tris HCl pH 8.0. The elution profile: 5 mL of a wash buffer containing 20 mM Tris HCl pH 8.0, 10% glycerol, 200 mM NaCl, 0.1% DDM, and 5 mM DTE; then, the protein was eluted with 3 mL of the same buffer containing 10 mM imidazole and 3 mL of the same buffer containing 50 mM imidazole; fractions of 1 mL were collected. The third fraction of protein eluted with 10 mM imidazole and the first and the second fraction of 50 mM imidazole were pulled together for subsequent desalting using PD-10 column using the desalting buffer composed of 20 mM Tris HCl pH 8.0, 0.1% DDM, 10% glycerol, and 5 mM DTE. The desalted protein was then used for reconstitution in proteoliposomes as described in the following paragraph. Protein concentration was estimated by the Chemidoc imaging system to calculate the *Sl*CAT2 specific activity as previously described [59]. 

### 4.4. Reconstitution of the SlCAT2 Transporter into Liposomes

The desalted *Sl*CAT2 was reconstituted by removing the detergent from mixed micelles containing detergent, protein, and phospholipids by incubation with Amberlite XAD-4 in a batch-wise procedure, as previously described [60]. The composition of the initial mixture used for reconstitution (except when differently indicated) was 400 µL of the purified protein (6 µg protein in 0.1% DDM), 80 µL of 10 % TX-100, 120 µL of 10% egg yolk phospholipids in the form of sonicated liposomes prepared as previously described [61], 15 mM ATP, and 10 mM Tris Hepes pH 7.5 (except where differently indicated) in a final volume of 700 µL. After vortexing, this mixture was incubated with 0.5 g Amberlite XAD-4 under rotatory stirring (1200 rpm) at 23 °C for 40 min in a batchwise procedure as previously pointed out [62].

### 4.5. Transport Measurements

Five-hundred-and-fifty microliters of proteoliposomes were passed through a Sephadex G-75 column (0.7 cm diameter × 15 cm height) preequilibrated with 10 mM Tris Hepes pH 7.5. From these columns, 550 µL of proteoliposomes were collected and divided into aliquots (samples) of 100 µL. Transport was started by adding 100 µM of [^3^H]Arg to the proteoliposome samples. The pH gradient experiments were performed reconstituting protein in liposome with 10 mM Tris Hepes pH 5.5. After incubation with Amberlite XAD 4 resin, 550 µL of proteoliposomes were passed through a Sephadex G-75 column preequilibrated with 0.5 mM Tris Hepes pH 5.5. Transport was started by adding 100 µM [^3^H]Arg buffered with 10 mM Tris Hepes at the different pH as indicated in the figure legend. 

The changes in external/internal osmolality were performed by adding in the extraliposomal/intraliposomal compartment appropriate concentrations of sucrose as indicated in the figure legend.

Transport reaction was stopped by adding 5 mM Pyridoxal Phosphate (PLP); according to the stop inhibitor method, the same inhibitor was added at time zero to control samples (blank) [63]. The initial rate was measured in 10 min, i.e., in the linear range of time courses as previously described [7]. The PLP insensitive radioactivity associated with the control samples (blank) was less than 30% with respect to the PLP-sensitive arginine transport.

For efflux measurements, proteoliposomes containing 15 mM ATP were preloaded by incubation with 100 µM [^3^H]Arg (1 µCi/mL) for 90 min [64]. External compounds were removed by another passage through Sephadex G-75 and efflux was measured as indicated in the figure legend. In both uptake and efflux, transport was stopped by passing 100 µL of each sample through a Sephadex G-75 column (0.6 cm diameter × 8 cm height) in order to separate the external from the internal radioactivity. Samples were eluted with 1 mL 50 mM NaCl and collected in 4 mL of scintillation mixture, vortexed and counted. 

The experimental values were corrected by subtracting the respective controls. Specific activity was calculated and expressed as nmol/mg at a given time of measurement or as nmol/(mg × min) in the case of initial rate measurement. For calculation of initial rate, transport was measured in 10 min, i.e., within the initial linear part of the uptake. Grafit 5.0.13 software (Erithacus Software, West Sussex, UK) was used to derive IC_50_ values in inhibition assays and to measure transport rate by first-order rate equation and by a single exponential decay with offset. 

### 4.6. Other Methods

CHS was solubilized in 20 mM Tris HCl pH 8.0 and 5% TX-100 by two sonication cycles of 2 min (no pulse, 40 W) with a Vibracell VCX-130 sonifier (SONICS, Newtown, CT, USA) as previously suggested [65]. Solubilized CHS was added to liposome preparation under rotatory stirring (1200 rpm) at 23 °C for 30 min. Electrophoresis was conducted using 12% SDS-PAGE and the resulting gel was stained using standard silver staining procedure. 

### 4.7. Homology Modeling and Docking Analysis

The crystal structure of *Gk*ApcT (5OQT) was used as a template to build the homology structural model of *Sl*CAT2 [41]. The amino acid sequence of *Sl*CAT2 and *Gk*ApcT were aligned through the Clustal Omega [66]. The alignment was used to run the program Swiss Model [67]. On the basis of the binding site of *Gk*ApcT, the position of Arg was determined using the software ArgusLab (M.A. Thompson, ArgusLab 4.0.1, Planaria Software LLC, Seattle, WA, USA). 

### 4.8. Statistical Analysis

Results were analyzed by nonparametric Student’s *t*-test or 1-way ANOVA test as appropriate as described in figure legends.

## Abbreviations

APC	Amino acid Polyamine Choline
*Sl*CAT2	*Solanum lycopersicum* CAT2
*Gk*ApcT	*Geobacillus kaustophilus* ApcT
CAT	Cationic Amino acid Transporter
CRAC	Cholesterol Recognition/interaction Amino acid Consensus sequence
TX-100	Triton X-100
DTE	DiThioErythritol
CHS	Cholesteryl HemiSuccinate
DDM	n-Dodecyl-β-d-Maltoside
